# Traditional Use of Wild and Domestic Fauna among Different Ethnic Groups in the Western Himalayas—A Cross Cultural Analysis

**DOI:** 10.3390/ani12172276

**Published:** 2022-09-02

**Authors:** Musheerul Hassan, Shiekh Marifatul Haq, Riyaz Ahmad, Muhammad Majeed, Hakim Ali Sahito, Madeeha Shirani, Iqra Mubeen, Muhammad Abdul Aziz, Andrea Pieroni, Rainer W. Bussmann, Abed Alataway, Ahmed Z. Dewidar, Mohamed Al-Yafrsi, Hosam O. Elansary, Kowiyou Yessoufou

**Affiliations:** 1 Clybay Research Private Limited, Bangalore 560114, India; 2 Department of Ethnobotany, Institute of Botany, Ilia State University, 0105 Tbilisi, Georgia; 3 Wildlife Crime Control Division, Wildlife Trust of India, Noida 201301, India; 4 Department of Botany, University of Gujrat, Hafiz Hayat Campus, Gujrat 50700, Pakistan; 5 Department of Zoology, Shah Abdul Latif University, Khairpur 66020, Pakistan; 6 Department of Zoology, Government College University Lahore, Lahore 54000, Pakistan; 7 Department of Environmental Sciences, Informatics and Statistics, Ca’ Foscari University of Venice, Via Torino 155, 30172 Venice, Italy; 8 Department of Medical Analysis, Tishk International University, Erbil 44001, Iraq; 9 University of Gastronomic Sciences of Pollenzo, Piazza V. Emanuele II 9, Pollenzo, 12042 Bra, Italy; 10 Prince Sultan Bin Abdulaziz International Prize for Water Chair, Prince Sultan Institute for Environmental, Water and Desert Research, King Saud University, Riyadh 11451, Saudi Arabia; 11 Department of Agricultural Engineering, College of Food and Agriculture Sciences, King Saud University, Riyadh 11451, Saudi Arabia; 12 Plant Production Department, College of Food & Agriculture Sciences, King Saud University, Riyadh 11451, Saudi Arabia; 13 Department of Geography, Environmental Management, and Energy Studies, University of Johannesburg, APK Campus, Johannesburg 2006, South Africa

**Keywords:** ethnic groups, wild fauna, western Himalayas, cross-culture, ethnozoology

## Abstract

**Simple Summary:**

In traditional medicinal systems, animals play an essential role in treating health issues (zoo therapy) as different body parts are used to treat different diseases. Meanwhile, local traditional knowledge (TK) is an important aspect of cultural legacy that can depict the relationship between communities and nature. Recently ethnobiologists have focused on cross-cultural research in order to document and measure the processes that govern the evolution of traditional knowledge within a culture, as well as to use it in the future. In the present study, we documented ethnozoological knowledge across eight ethnic groups in the Jammu and Kashmir Himalayas. Comparative analysis indicated that Balti and Brokapa were more closely related groups due to high overlap (N = 7) of the use of medicinal species. A total of thirteen idiosyncratic species were recorded for the Kashmiri ethnic group, followed by two idiosyncratic species (*Anser indicus*, *Perdix hodgsoniae*) for Balti and two idiosyncratic species (*Capra aegagrus hircus*, *Cuon alpinus*) for Changapa. The Pearson correlation coefficient supported the strength and direction of a link between ethnic groups and regions. Cluster analyses revealed two primary clusters of the 79 animal species recorded for eight ethnozoological uses based on fauna similarity. Furthermore, all ethnic groups primarily used the fauna for medicinal and food purposes. Chest infections were frequently treated by the maximum number of species (N = 9), followed by paralysis by seven species. The current ethnozoological study provides needed information such as cross-cultural traditional knowledge of medicine, food, and religious value; combining ethnic knowledge with a scientific approach can make a significant contribution to the long-term development of native communities.

**Abstract:**

Animal-derived products have an important role in treating many health conditions and have widely been used across cultures. In South Asia, ethnozoological research has been conducted only by a small number of researchers. Therefore, this area of research needs further exploration in order to preserve the eroding ethnozoological knowledge of medicinal animals severely affected by ongoing social change. This study was conducted in the region of Jammu and Kashmir from February 2019 to August 2021. The study was carried out among eight different ethnic groups living in the region. A total of 374 informants were selected and data were collected through semi-structured interviews and verified through group discussions. Data was analyzed using different statistical tools, including R 4.0.0. The cross-cultural data were compared through Bioinformatics and Evolutionary Genomics software and later subjected to further analysis, applying Pearson correlation and ordination techniques (Principal Component Analysis). We recorded a total of 79 animal species being used by the eight studied ethnic groups in the region. Wild animal species were mainly used for therapeutic purposes. Chest infections, sexual problems, and paralysis were frequently treated diseases. Flesh was the most commonly part used. The cross-cultural comparison showed a remarkable heterogeneity in the use of the animals among the different groups, which could be an effect to the historical sociocultural stratifications, as well as different religious affiliation of certain groups preventing them to forage or hunt certain animals. Some groups however showed prominent overlap of uses of some recorded species. For instance, *Lerwalerwa* and *Bubalus bubalis* were commonly used by both Gujjar and Pahari, which could be referred to the fact that they have gone through significant socio-cultural contact, and they are exogamous to each other. The Pearson correlation coefficient supported the strength and direction of an association between ethnic groups and regions. The study makes an important contribution to the field of ethnozoology in the Himalayas by providing insights to understand the historical human and nature relationships and supplying a baseline for developing future conservation efforts in the region to protect the wild fauna

## 1. Introduction

Between ethnic communities, there is normally a variation of the usage of animal species due to their cultural legacy [1]. Environmental and economic factors also determine the usage of species [2]. Local and indigenous communities often use a variety of species for their survival, highlighting the vital role of fauna in their lives [3]. The use of animals is not limited to food and medicine, but includes entertainment, magic, religion, trade, and tools [4,5,6,7,8] all throughout human history, e.g., reflected in cave wall paintings [9,10]. The knowledge of using particular species for a particular purpose is obtained over time and passed onto future generations [10]. Endemic traditional knowledge is an important facet of cultural heritage, indicating a close association between people and nature [9]. Almost 8.7% of important chemical compounds of allopathic medicine are obtained from animals [11,12,13]. Despite this importance, plants are still prioritized over animals [14]. In traditional Chinese medicine, 1500 animal species are used, and in Unani medicine, 500 species, denoting the importance of fauna in traditional medicinal systems [15]. India has a great diversity of animal species, harboring almost 10% of the global fauna, and ranking first in terms of insect diversity, followed by fish, birds, reptiles, mammals, and amphibians [15]. In India, about 70% of the rural population lacks modern healthcare facilities and thus depend on traditional medicinal systems [15]. Due to factors such as urbanization and industrialization, these communities are losing socioeconomic and cultural characteristics, including traditional medicinal knowledge. In this regard, it becomes imperative to document this traditional knowledge [16,17,18,19,20]. The exploration and documentation of indigenous knowledge is vital to obtain first-hand information about the different usage of animals [21,22,23]. Moreover, with the growing challenge of resistance of pathogens against allopathic medicine, there is a dire need to investigate new combinations of natural constituents with additive and synergistic effects [24,25]. According to Leonti and Casu [20], the documentation of ancestral knowledge in ethno-biological surveys may cover the existing gap to discover effective drugs. The erstwhile state of Jammu and Kashmir is well known for its rich diversity due to its topographical, altitudinal, and geographical variation [26]. Various ethnic communities live in this potential traditional knowledge area [27,28]. The present study focused on (1) documentation of the medicinal uses of wild and domestic birds and mammals among the local communities in the study region. (2) Comparison of the recorded data from different cultural groups in order to understand the socio-cultural connections that have influenced the cultural knowledge of individuals from each ethnic group. This is the first cross-cultural ethnozoological study carried out in the region. We hope that the results of the study will provide baseline data for future conservation programs in the region and that policy makers would pay attention to preserve the cultural heritage and protect the wild fauna of the region.

## 2. Materials and Methods

### 2.1. Study Area

**Figure 1:**
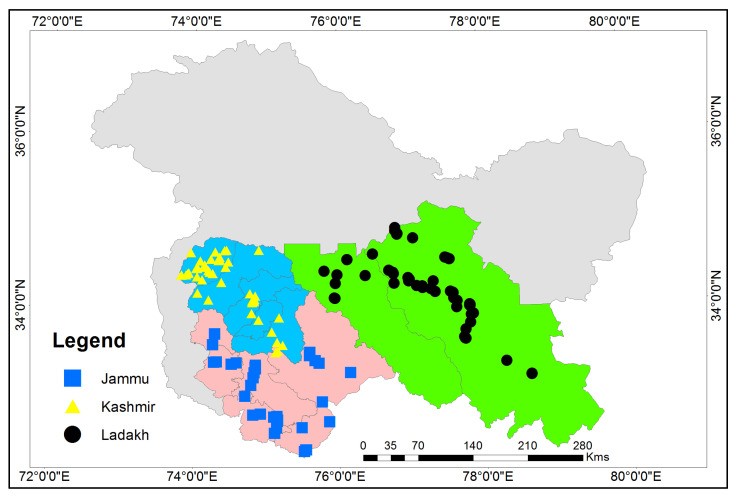
Map of the Jammu and Kashmir (J and K) and point showing the sampling sites in erstwhile states of Jammu and Kashmir.

Jammu and Kashmir (Figure 1), former princely states of India, were recently separated into two union territories (Jammu & Kashmir and Ladakh) by the Central Government of India (https://egazette.nic.in/WriteReadData/2019/210407.pdf) (accessed on 17 January 2021). The region is bordered to the northeast by the Uygur Autonomous Region of Xinjiang (China), to the east by the Tibet Autonomous Region (China), and the Chinese-administered portions of Kashmir, to the south by the Indian states of Himachal Pradesh and Punjab, to the southwest by Pakistan, and the northwest by the Pakistani-administered portion of Kashmir. Geographically, Jammu and Kashmir comprise rugged mountains and barren slopes. Based on the Koppen classification, Jammu and Kashmir have a Dfb (Warm-summer humid continental climate) where as Ladakh has a Dwd climate (Monsoon-influenced extremely cold subarctic climate) [29,30]. The environmental conditions of Jammu are not uniform, i.e., the region comprises sub-tropical plains with hot summers and cold winters with monsoonal climes and is highlands temperate. The region of Kashmir is temperate with warm summers and cold winters. Ladakh is a high altitude cold arid region deprived of vegetation. As a result, the availability of resources is varied and distinct, especially that which lies between the trans-Himalayan Ladakh region and the more sub-tropical and temperate Jammu and Kashmir. Jammu and Kashmir also harbor rich ethnic and cultural diversity. According to the 2011 Census (https://uidai.gov.in/images/state-wise-aadhaar-saturation.pdf) (accessed on 17 January 2021), the population of Jammu and Kashmir was 13,606,320, divided into Muslims (67% of the population), Hindus (about 30%), Sikhs (2%), and Buddhists (1%).

The rich wild fauna diversity of J and K has enormous economic potential. Important wild fauna includes the last viable population of the threatened hangul (*Cervus hanglu*), musk deer (*Moschus cupreus*), markhor (*Capra falconeri*), Himalayan tahr (*Hemitragus jemlahicus*), and other species of mammals including Himalayan brown bear (*Ursus arctos*), common red fox (*Vulpes vulpes*), snow leopard (*Panthera uncia*), and Tibetan argali (*Ovis ammon*) [26,31]. The region also harbors an enormous source of domestic fauna, e.g., *Bos grunniens*(Yak)*, Capra aegagrus hircus* (Pashmina goat), *Bos taurus primigenius* (Cow) and other species like, and *Anas platyrhynchos domesticus* (Duck) [32].

### 2.2. Field Study

The present study was based on a field survey conducted among the different ethnic groups, such as the Pahari, Gujjar, Bakarwal, Dogra, Kashmiri, Brokpa, Balti, and Changapa, following different faiths, such as Islam, Hinduism, Sikhism, and Buddhism, as recorded across the study area (Table 1). Prior to each interview, verbal consent was obtained from the participants and the Code of Ethics adopted by the international Society of Ethno-biology was followed [33]. Random sampling was used to gather information using semi-structured and open-ended discussions [26]. We interviewed 374 respondents, among which 216 were men and 158 were women (Table 1). A total of 25 field visits during the survey were made. Mammals and birds were identified using the field guides and following the literature [34,35,36,37,38]. Taxonomic identification was carried out by using the online database “Integrated Taxonomic Information System” (https://www.itis.gov) (accessed on 17 August 2021) and using the regional literature [26,31]. We have also compared the recorded data with the previous ethnobiological literature carried out in the nearby regions, particularly surrounding the Himalayan regions [10,26,32,39,40,41,42,43,44,45]. The conservation assessment of documented fauna species was done as per the International Union for Conservation of Nature (IUCN, 2019) Red List (www.iucnredlist.org/) (accessed on 17 August 2021) and used the regional literature [31].

### 2.3. Socio Economic Background

Most of the ethnic groups in the study area are associated with agriculture, livestock, and allied services [32]. Kashmiri and Dogra are associated with agriculture and horticulture, and many are government job holders, shopkeepers, daily wage laborers, and craftsmen. Gujjar and Bakarwal are mostly linked with livestock rearing of a nomadic nature, Balti are associated with cattle rearing and horticulture, Brokapa are related with cattle rearing and horticulture and many are wage laborers, Changapa are mostly nomadic associated with goats. Most of the Kashmiri live in the valley (Kashmir), Dogra mostly live in Jammu, Gujjar and Bakarwal have populated Kishtawar, Rajouri of Jammu region, however, they also tend migrate to the valley of Kashmir in summer for grazing their livestock, whereas Balti, Changapa, and Brokapa live in the Ladakh region. The Changapa also known as Champa are semi-nomadic and mainly seen in the Changtang area. Gujjars are a large heterogeneous group mostly found in hilly areas, and are dependent on nature, and the most important inhabiting areas from Kashmir include “Karnah, Keran, and Tangdaar”, and from Jammu, “Poonch, Rajouri, and Kishtawar” are notable sites. These people are unique in their culture with a potential traditional knowledge. Pahari live in lower Himalayas and are also found in hilly areas, often close to Gujjar, and many of them are also found in low lying areas (near to Kashmiri). The Kashmiri are mostly seen in the Kashmir and are engaged in rapid urbanization; hence, people here are less dependent on nature, however, they are still associated with agriculture, of which paddy cultivation is the prime concern. Bakarwal are nomads and herd in high-altitude regions. They migrate across Jammu and Kashmir with respect to season, and during the maximum time of year they can be seen in the Rajouri and Kishtawar area. These people have a command of the origin of flora and fauna across the western Himalayas as they are totally dependent on their livestock and nature. Dogra are mostly found in the areas such as Reasi, Kathua, and Sambha, in the Jammu region. Balti are located in Ladakh, especially in the Kargil district, but also live in Leh. Brokapa also inhabit Ladakh. Changapa are high-altitude pastoralists inhabiting the Changtang region, raising goats, the highly pedigreed and prized Changra goat that yields luxurious pashmina fibers.

### 2.4. Data Analysis

Data was analyzed using Principal Component Analysis (PCA) [26]. PCA was used to find hypothetical variables (components) that account for as much of the variance in our multi-dimensional data as possible. For that, we used a matrix of presence/absence of animal species in each of the ethnic groups in the three regions studied and calculated the singular valued composition of the (centered and possibly scaled) data matrix. PCA was done using the Software R Studio 4.0.1. With PCA, we elucidated how each, or a set of species, were related to each region and ethnic groups evaluated. Bioinformatics and Evolutionary Genomics software were used to conduct cross-cultural comparisons between ethnic groups [46,47]. We computed the correlation coefficient between the ethnic groups (Pahari, Gujjar, Kashmiri, Dogra, Bakarwal, Brokpa, Balti, and Changapa) and regions using the Pearson method. Results were plotted in a correlogram [48] with the corrplot package [49].

## 3. Results

### 3.1. Ethnozoological Inventory

The current study recorded 79 animal species used by ethnic people. The study is the first detailed survey on the use of wild and domesticated animals and birds used in local medicines and other ethno-uses in the Himalayan region. Data were grouped into two categories, i.e., domestic (N = 14) and wild (N = 65), and in both groups (birds, mammals). The domestic species belonged to five families, i.e., Bovidae (N = 7) was the dominant family, followed by Anatidae, Camelidae, Equidae (N = 2 each), and Phasianidae (N = 1). Wild species were scattered into 23 families in which Anatidae (N = 12) was the dominant family, followed by Phasianidae (N = 11) and Bovidae (N = 10). Data presented here for each of the quoted species along with its zoological name, local name, zoological family, parts used, and medicinal uses are provided in Table 2. Results reveal that the local people have frequently reported wild animal species over the domestic for medicinal uses. This can be attributed to the belief of ethnic communities in traditional medical systems that prioritize wild fauna over domestic. Similar use of animals for zoo-therapy and other ethno-zoological purposes was described by Verma et al. [50] from Assam, India, and Castillo and Ladio [51] in Argentina. Barbosa et al. [52] reported the dominance with respect to ethnozoological usage of wild fauna over domestic from the north-eastern area of Brazil; furthermore, the usage ascendency of wild fauna is common among ethnic communities at the global level [53,54].

**Figure 2:**
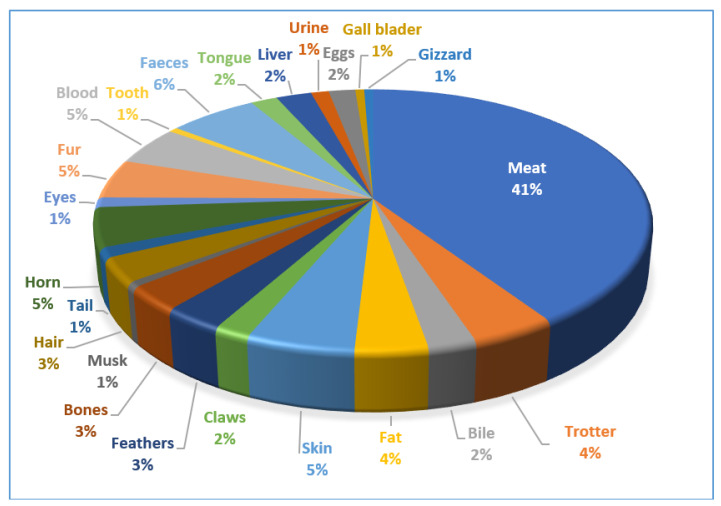
Percentage of body parts used by ethnic communities for ethnozoological practices.

Findings showed that that meat (41%) was the dominant part used by the local communities for medicinal purposes followed by feces (6%), fur (5%), skin (5%), horn (5%), blood (5%), trotters (4%), feathers (3%), hair (3%), bile (2%), claws (2%), tongue (2%), liver (2%), eggs (2%), gizzard (1%), gallbladder (1%), urine (1%), teeth (1%), eyes (1%), tail (1%), and musk (1%) (Figure 2). All the reported medicinal species (domestic, wild) were found in the study area, and many bird species were migratory. Local people hunted wild species for medicinal as well as for food and other uses. It is important to note that the Dogra community has to follow certain religious obligations and they tend to avoid hunting, whereas the rest of the ethnic groups have always been engaged in hunting and foraging processes. The documented species play a vital role in traditional medicinal systems for the Unani, Ayurveda, and Sowa-Rigpa, who employ different parts of the species for variety of ailments. It is believed that home therapies are more efficient in treating various kinds of health. In the study area, traditional use of home-based remedies is associated with two kinds of belief systems, known as (a) *Saad gaza* and (b) *Garailov alaj. Saad gazza* simply means “simple diet” and people believe that a good and simple diet is itself a medicinal food and keeps diseases away. Similarly, *Garailov alaj* consists of medicinal remedies obtained from different animals at the home level and used to treat primary health disorders. It is also relevant to mention that although hunting has been banned by the government, people still poach wild animals for its use as food and for medicinal purposes whenever they need. It is also interesting that hunting is relatively easy in the winter season, as wild animals come out from their habitats and go around to seek food and, therefore, the hunter has the maximum possibility to find them.

### 3.2. Medicinal Use of Documented Species

**Figure 3:**
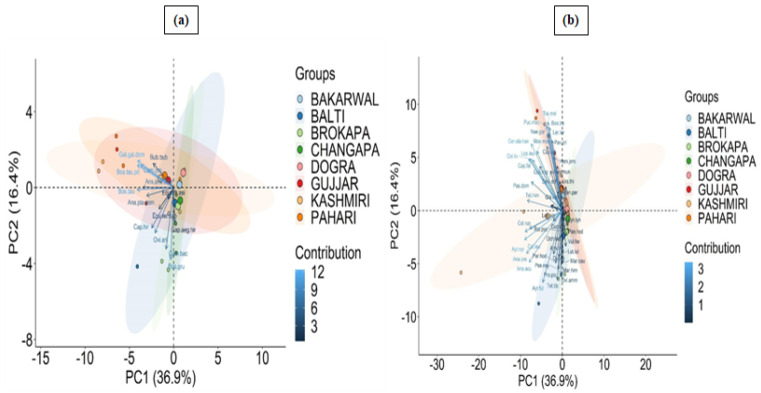
PCA diagram representing clustering of (**a**) wild; (**b**) domestic species among ethical groups. The complete name of the species is shown in Table 2.

The documented medicinal species (wild/domestic) were used to treat different diseases. Wild species were used for 29 diseases, which included chest infections, sexual diseases, paralysis, ulcer, arthritis, body weakness, cancer, cataract, gynecological diseases, wounds, urinary diseases, gout, epilepsy, plague, inflammation, liver diseases, hemorrhoids, tuberculosis, dermatitis, leprosy, heart diseases, eye diseases, diabetes, blood purifier, foot burning issues, fracture, pain, diarrhea, and cold (Table 2). Chest infections were frequently treated by the most number (N = 9) of species, i.e., *Capra sibirica hemalayanus*, *Ovis aries vignei*, *Panthera uncia*, *Ursus arctos*, *Columba leuconota*, *Columba livia*, *Lerwa lerwa*, *Pucrasia macrolopha*, and *Tetraogallus himalayensis*, followed by paralysis treated by seven species (*Macaca mulatta*, *Ursus arctos*, *Ursus thibetanus*, *Alectoris chukar*, *Columba leuconota*, *Columba livia*, *Tetraogallus tibetanus*). In the study area, winter is very cold, therefore, it could be attributed to the fact that the major categories of diseases were chest infections (i.e., cough, cold). Sexual diseases were commonly treated by the ingredients derived from *Lutra lutra*, *Moschus moschiferus*, *Mareca Penelope*, and *Perdix hodgsoniae* (Table 2). The PCA revealed significant variance between use of species and ethnic groups, with certain groups of species being more associated to one ethical group than another (Figure 3a,b). For example, dermatitis is treated by the urine of *Equus africanus asinus* in Gujjar and Bakarwal; similarly, skinned off and sundried tongue of *Cuon alpinus* is used to treat ulcers in Changapa (Table 2). PC1 and PC2 explain percentages of the total variation in the species distribution in the biplot, in which species grouped in clusters are closely correlated to those particularly ethnic groups. A biplot shows how the species in the PCA relate to one another (which samples are similar and which are distinct) while also revealing how each variable contributes to each principle component.

Machkour et al. [26] from the Himalayas reported the use of fauna species across ethnic communities in Mexico for medicinal usage (zootherapy).Jugli et al. [55] reported the use of different wild animals and birds such as *Ursus arctos* for toothache and *Columba livia* for weakness across two ethnic groups (*Tangsa* and *Wancho*) from North-East India. Altaf et al. [39] reported the use of *Hystrix indica* for treating skin infections, rheumatic pains, *Rattus rattus*for convulsions, joint pain, and wound healing, across local ethnic communities from Punjab Pakistan. Dhakal et al. [44] reported the use of *Muntiacus muntjac* for overcoming constipation and food poisoning, *Ursus thibetanus* for fever, liver disorders, heart diseases, and body ache, *Vulpes* for joint pain, *Hemitragus jemlahicus* for dysentery in local communities from the Sikkim Himalayas in India. Negi and Kandari [43] reported the use of *Pseudois nayaur* for stomach pain and fever, *Canis familiaris* for epilepsy, skin diseases, and earache, *Panthera pardus* for weakness, body pain, and sexual stimulant, and *Macaca malatta* for asthma and rheumatism from the Bhotiya tribe from Uttarakhand, India. 

Results also showed that only 13 diseases, i.e., chest infections, sexual issues, ulcers, arthritis, body weakness, cracked heals, cyst, gynecological issues, wounds, chilblains, dandruff, and dermatitis, were treated with parts obtained from domestic fauna (Table 2). Among the enlisted diseases, sexual issues were treated with the highest number (N = 5) of species (*Camelus bactrianus*, *Camelus dromedaries*, *Anser anser domesticus*, *Gallus gallus domesticus*, *Anas platyrhynchos domesticus*) followed by arthritis using three species (*Bos grunniens*, *Camelus bactrianus*, *Camelus dromedaries*). Dermatitis was treated using two species (*Equus ferus caballus* and *Equus africanus asinus*). Altaf et al. [39] reported the use of different domestic species to overcome weakness, to sharpen memory, and as an antidote and sexual stimulant in Pakistan. Similarly, Jugli et al. [55] reported the use of species such as *Gallus gallus domesticus* for body burns, *Capra hircus* for early detachment of the umbilical cord in the Indian Himalayas. Castillo and Ladio [51] reported the use of *Capra aegagrus hircus* to treat empacho (indigestion), and *Gallus gallus domesticus* for burns in Argentina. Other similar studies which are in accordance include Mahawar and Jaroli [56] and Quave et al. [57]. It is very important to mention that medicinal purposes are restricted to a particular number of fauna species, in addition, for the treatment of diseases, plants are used often over animals due to diversity, wide availability, and easy collection.

### 3.3. Cross Cultural Comparison

Cross cultural comparisons (Table 3) of the recorded medicinal uses for the quoted species showed that only one species (*Capra hircus*) was used among all the ethnic groups. This is due to the fact that said species is domestic and, hence, is artificially implanted. Additionally, it is widely distributed in the western Himalayan regions and survives in all weather conditions. It is also referred to as the poor man’s buffalo due to easy rearing and low cost of survival when compared to other animals. We found remarkable variations (mosaic pattern) in the use of the other reported medicinal species, however (Table 3). Comparative analysis indicated that Balti and Brokapa were more closely related groups due to high overlap (N = 7) of the use of species with medicinal properties (*Athene noctua*, *Marmota himalayana*, *Tetraogallus tibetanus*, *Ovis ariesvignei*, *Camelus bactrianus*, *Vulpes ferrilata*, *Lutra lutra*). This high overlap could be attributed to the same geographical location which has made it possible to have equal access to the resources under same socio-ecological conditions. Both groups are exogamous with each other (Table 1). The use of species was affected by religious affiliation, e.g., Brokapa did not use dairy and poultry because of religious taboos. Their economic condition is also not stable as most are wage laborers. In spite of the prevailing laws (wildlife protection act 1972; https://legislative.gov.in/sites/default/files/A1972-53_0.pdf (accessed on 17 August 2021)), this economic compulsion drives the population to use wild species for good earning, medicine, and food. In the Balti group, pious people were reluctant to use food and medicine obtained from wild animals. They believed that being wild made things impure and they contained wildness which could affect their piousness. It is important to note that a small number of people from both Balti and Brokapa were associated with camels (*Camelus bactrianus*) in the tourism industry. The use of camels is only during the summer and is halted during the winter due to heavy snowfall, which prevents the tourists from coming. In Changapa, although the prime source of medicine was animals (wild), killing fauna species for food is prohibited due to religious obligations; meanwhile, some use animals as food as long as the animals are not killed/slaughtered/poached for them. The prime source of food from animals is milk, which is mostly obtained from goats. *Capra aegagrus hircus* is a unique goat reared only by this ethnic group.

A maximum of thirteen idiosyncratic uses for certain species i.e., *Porphyrio poliocephalus*, *Hystrix indica*, *Streptopelia decaocto*, *Ardeola grayii*, *Cygnus Columbianus*, *Milvus migrans*, *Nettarufina*, *Anas poecilorhyncha*, *Spatula clypeata*, *Mareca penelope*, *Arborophila torqueola*, *Mareca Strepera*, and *Aythya ferina* were recorded for the Kashmiri ethnic group. This is due to the reason that the valley of Kashmir shows a temperate climate with warm summers and a cold winter, which lures some diverse migrant water bird species from other parts of the world. These bird species are traditionally used for a variety of purposes, and can be easily seen in local water bodies i.e., (Dal lake, Wular lake, Nageen lake) surrounded (inhabited) by Kashmiri people. Only two idiosyncratic species (*Anser indicus*, *Perdix hodgsoniae*) were recorded for Balti and two idiosyncratic species (*Capra aegagrus hircus*, *Cuon alpinus*) for Changapa. The possible reason for the fewer number of idiosyncratic species is that both ethnic groups (Balti and Changapa) inhabit a high- altitude, cold, and arid region (Ladakh) that is deprived of vegetation and has a low diversity of species. Further, from the Table 3, it is quite clear that the Balti, Brokapa, and Changapa groups are more related to each other concerning species utilization, forming a group with most similarities. The same is true with the Gujjar, Bakarwal, Kashmir, and Pahari groups; meanwhile, both groups show dissimilarities regarding species usage.

**Figure 4:**
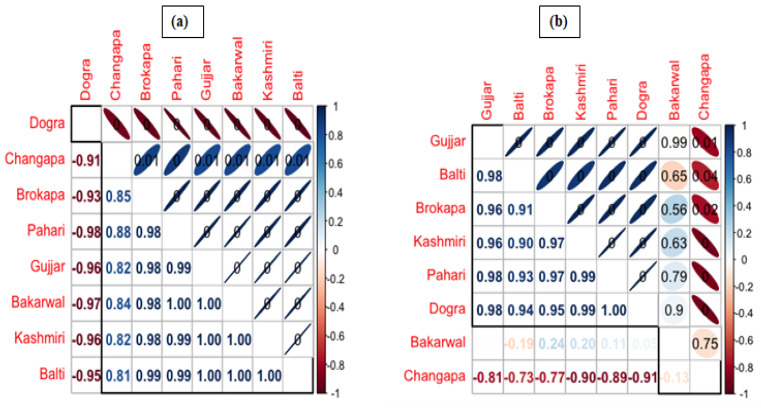
Correlogram showing the Pearson correlation results between ethnic groups evaluated in the 3 regions for (**a**) wild and (**b**) domestic animals.

The Pearson correlation coefficient further underlined the strength and direction of the association between ethnic groups (Figure 4a,b). The *p*-values are displayed at the top, and the Pearson correlation coefficients are displayed at the bottom (Figure 4a,b). Our findings are in accordance with Solanki and Chutia [58] from India who reported the diversity of use pattern of fauna across different ethnic groups. Similarly, Solavan et al. [59] reported the use of fauna across different ethnic communities in Tamil Nadu, India. Mahawar and Jaroli [56] revealed the use of animal fauna by different indigenous communities around Ranthambore National Park, India. Various ethnozoological studies by Haq et al. [60,61], Alves et al. [62], and Santos et al. [63] also used a quantitative ethnobiological approach in their studies.

### 3.4. Comparison with Other Ethnobiological Studies in the Nearby Regions

**Figure 5:**
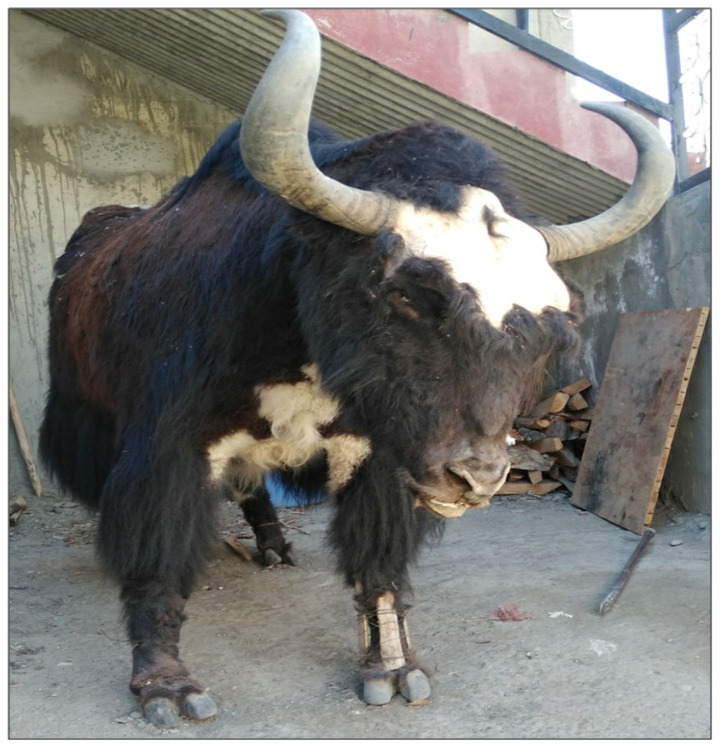
Taxidermied yak in Ladakh.

Literature comparisons have shown that some of the uses of recorded species were new to the ethnobiological literature, like, the liver of *Marmota caudata* is used to treat bone weakness, bones of *Vulpes ferrilata* to treat lung ulcers, back pain, and rheumatic pain, pelts of *Rattus pyctoris* to treat urinary incontinence, the flesh of *Passer domesticus, Lerwa lerwa* and *Streptopelia decaocto* for cardiac issues, common cold, and asthma, respectively. Powdered eggshells of *Mareca Penelope* were used to treat infertility in males. Young *Aythya nyroca* were cooked and eaten to gain strength after delivery. Fat of *Aythya fuligula* was used to treat neck pain and long feathers from wings used to overcome nightmares. The liver, kidneys, head, and tongue of *Ovis aries* were used to perform black magic, and likewise, the eyes, feathers, and blood of *Milvus migrans* are also used in black magic. The urine of *Equus ferus caballus* and *Equus africanus asinus* is used to treat dermatitis, dung of *Equus ferus caballus* to extract the larvae of pathogens from wounds, and hair from the tail was used to remove cysts developed on the skin. In addition to ethnozoological applications among the bio-geographic regions, we also found the practice of taxidermy (art in the preservation and restoration of dead specimens for long term storage and display). In Kashmir and Jammu, people take advantage of this technique during the time when a calf of a cow dies, and the cow is reluctant to give milk. The skin of calf is knifed out and mounted around straw to look like a new calf, allowing the owner to milk the cow easily. Meanwhile, in Ladakh, people restore slaughtered or dead yak (Figure 5) by stuffing the skin with straw. The obtained specimen is kept in hotel galleries as an art display and decoration.

### 3.5. Conservation of Species

To understand the background of the relationship between mankind and natural resources is very important for the drafting of fauna conservation strategies [64]. In this regard, ethnozoology provides required information such as traditional knowledge of medicine, food, and culture, and hence, make a significant contribution [65,66,67,68]. In order to maintain wild species survival while continuing to meet global demand for biological resources, it is vital to identify and manage the implications of hazards associated with their use. Local people rely on wild species for medicine and food, and they have cultural and religious value as well. Various animal species have become a favorite target due to their use by indigenous communities in traditional systems of medicines and other uses resulting in the decline of species, which puts them into different IUCN categories (Table 2). We concluded that tribal groups constitute a major threat to fauna species because of a lack of modern services (medical, education), and the communities are typically disadvantaged owing to economic limitations. The fauna species in the respective place become an easy target for these tribes, such as bear (*Ursus thibetanus)* being killed for bile for the treatment of jaundice, Markhor (*Capra falconeri*) being slaughtered for meat and trophies, and Kashmiri stag (*Cervus hanglu*) being poached for food, medicine, and trophies. There is an urgent need to address the issue by implementing a community-based conservation (CBC) development programme that will include the government, non-governmental organizations (NGOs), and citizens who have been impacted by wildlife (tribal communities and other communities), and other interested groups. This initiative will not only safeguard wild fauna, but it will also solve the challenges that local ethnic communities confront (which entice and drive them to rely on the wild). The best example of CBC comes from Tanzania, where wildlife species were protected while local populations were addressed [69].

## 4. Conclusions

This study emphasizes the importance of ethnozoological uses among the different ethnic groups in the erstwhile states of Jammu and Kashmir. The uses varied by ethnic groups, and the reliance on wildlife was consistent across the board. Furthermore, all ethnic groups exploited the fauna primarily for medicinal and food purposes. However, there is little precise documentation of such fascinating traditional knowledge in the study area. Over time, modern progress has accelerated and these traditional processes may be negatively impacted, threatening their survival in the foreseeable future. As a result, serious efforts must be made to preserve traditional knowledge as well as threatened wild species. Meanwhile, the present study could be valuable for investigations on pharmacological profile, and in-vitro and in-vivo investigations of biological compounds from the documented fauna could be interesting for the development of novel animal-based drugs to treat various health disorders. It is important to mention that we compared the ethnic groups on the use of fauna species; however, the effect of different ecological challenges in which the ethnic communities inhabit cannot be neglected, hence comparing the usage of species with different ecological requirements creates an indirect limitation of the present study.

## Figures and Tables

**Table 1:** Demographic status of the respondents from the study area.

Demographic Features	Total Percentage	Ethnic Groups (Erstwhile Jammu & Kashmir)							
		Kashmiri	Pahari	Bakarwal	Gujjar	Dogra	Balti	Brokapa	Changapa
Regions		Kashmir	Kashmir Jammu	Kashmir Jammu	Kashmir Jammu	Jammu	Ladakh	Ladakh	Ladakh
Respondents	374	61 (16.31%)	53 (14.28%)	47 (12.56%)	54 (14.43%)	47 (12.66%)	39 (10.42%)	37 (9.89%)	36 (9.62%)
Gender									
Male	216 (57.75%)	33	30	22	33	24	26	26	22
Female	158 (42.24%)	28	23	25	21	23	13	11	14
Age range (27–75)		average age	average age	average age	average age	average age	average age	average age	average age
Approx. number of inhabitants		6,797,587	1,000,000	200,000	800,000	250,000	145,000	48,439	1500
Original language		Kashmiri	Pahari	Gujari	Gujari	Dogri	Balti	Brokpake	Changthang/Byangskat
Religion		Shia and Sunni Islam Sikhism	Shia and Sunni Islam Hinduism	Sunni Islam	Sunni Islam Hinduism	Hinduism	Shia Islam Buddhism	Sunni Islam Buddhism	Buddhism
Marriages		exogamous with other muslims (Pahari) endogamous (Sikh)	exogamous with other muslims (kashmiri, gujjar) endogamous (Hindu)	exogamous with other muslims (Gujjar)	exogamous with other muslims (Bakarwal)	endogamous	exogamous with other muslims (Brokapa) exogamous with other buddhists (Brokapa)	exogamous with other muslims (Balti) exogamous with other buddhists (Balti)	endogamous
Livelihood source		horticulture and cattle rearing	horticulture and cattle rearing	pastoralism	horticulture pastoralism	horticulture and cattle rearing	cattle rearing horticulture	cattle rearing wage labor	pastoralism
Origin		indo-europena ethno-linguistic group	indigenous group of Himalayas	gurjara kingdom (570ce)	gurjara kingdom (570ce)	ikshvaku (solar) dynasty of northern india	tibetan descents	tibetan descents	tibetan descents

**Table 2:** Ethnozoological inventory of documented species.

Species (Family) (Abbreviation)	Local Name (English Name)	Parts Used	Ethno-Zoological Usage	Zootherapy	IUCN Status	Ethnic Groups
Wild Mammals						
*Boselaphus tragocamelus* Pallas, 1766 (Bovidae) (Bos.tra)	Nilgai * (Blue bull)	Flesh Dung	Flesh is cooked and consumed as food.	Dung obtained in the morning is painted to treat leprosy.	Least concern	Gujjar Pahari Bakarwal Dogra
*Canis lupus* Linnaeus, 1758 (Canidae) (Can.tra)	Shangku * (Grey wolf)	Flesh Tongue Stomach Blood	Flesh is made into amulets to ward off evil.	Flesh is cooked without oil to treat epilepsy and plague. Tongue, stomach, and blood are cooked without oil to cure inflammation, to increase digestion and treat diabetes, respectively.	Least concern	Balti Brokapa Changapa
*Capra falconeri* Wagner 1839 (Bovidae) (Cap.fal)	Markhor *	Flesh Horns	Flesh is cooked and consumed as food; meanwhile, long and spiraling horns are used as trophies.	--------------------------	Near Threatened	Gujjar Pahari Bakarwal Kashmiri
*Capra sibirica hemalayanus* Pallas, 1776 (Bovidae) (Cap.sib.hem)	Skyin (B) Skein (Br) Skee (C) Badi bakri (G,Ba) Beada (P) (Asiatic ibex)	Flesh Horns Mouth foam	Flesh is cooked and consumed as food. Horn is used as a trophy and decoration. Foam from the mouth is used as poison.	Horns are powdered, taken with hot water to treat chest infections.	Least concern	Gujjar Pahari Bakarwal Balti Brokapa Changapa
*Cuon alpinus* Pallas, 1811 (Canidae) (Cuo.alp)	Foyia (C) (Asiatic Wild Dog)	Tongue	-------------------------------	Tongue is skinned off, sundried, grinded and mixed with seeds of fennel powder and used to treat ulcers.	Endangered	Changapa
*Cervus hanglu* Wagner, 1844. (Cervidae) (Cer.ela.han)	Hangul * (Kashmiri stag)	Flesh Antlers	Flesh is cooked and consumed as food. Antlers are used for decorative purposes.	Antlers are burned to ashes and taken orally to treat hematuria. Ashes mixed with water to form paste, applied to affected areas to treat fracture, ulcers and acute pain.	Critically Endangered	Gujjar Pahari Bakarwal Kashmiri
*Hemitragus jemlahicus* Hodgson, 1841 (Bovidae) (Hem.jem)	Karth (D) Jagla (G,B) (Himalayan Tahr)	Flesh Dung	Flesh is cooked and consumed as food. Dung is dried and used as fuel on new year, believed to overcome poverty	-------------------------------	Near Threatened	Gujjar Bakarwal Dogra
*Hystrix indica* Kerr, 1792 (Hystricidae) (Hys.ind)	Sag * (Porcupine)	Quills Bile	Quills and guard hairs are used for traditional decoration.	Bile obtained is dried mixed with cinnamon and mint and taken orally to treat tuberculosis.	Least Concern	Gujjar Pahari Kashmiri
*Lutra lutra* Linnaeus, 1758 (Mustelidae) (Lut.lut)	Chusaman * (Common Otter)	Liver	-------------------------------	Liver is boiled in water, washed, roasted and consumed with olive oil to treat reproductive disorders.	Near Threatened	Balti Brokapa
*Lepus oiostolus* Hodgson, 1840 (Leporidae) (Lep.oio)	Ribong (Br) Rebeg (B) Khargosh (G) (Hare)	Flesh Fur Skin Droppings	Flesh is cooked and consumed as food. Fur and skin are used to make caps and jackets. Droppingsare used as fertilizers and hare is also employed for recreational purposes.	Droppings are used to treat skin diseases.	Least Concern	Gujjar Balti Brokapa
*Lynx lynx isabellinus* Linnaeus, 1758 (Felidae) (Lyn lyn)	Yie (Br) Yet (C) Ban billa (P,B) (Himalayan lynx)	Flesh Fur	Beautiful fur is used for costume making. Dried flesh is also used as an amulet to ward off evil.	Flesh is sun dried and cooked with *Amaranthus blitum* tender shoots to overcome body weakness and arthritis.	Least Concern	Pahari Bakarwal Brokapa Changapa
*Marmota caudata* Geoffroy, 1844 (Sciuridae) (Mar.cau)	Phia (Br) Faia (B) Fa (C) (Golden marmot)	Flesh Liver	Flesh is cooked and consumed as food.	Liver is boiled in water with a little vinegar, finely chopped, cooked with pigeon eggs, used to treat bone weakness.	Least Concern	Balti Brokapa Changapa
*Marmota himalayana* Hodgson, 1841 (Sciuridae) (Mar.him)	Phia (Br) Faia (B) (Himalayan Marmot)	Flesh Liver	Flesh is cooked and consumed as food.	Liver is boiled in water with a little vinegar, finely chopped, cooked with pigeon eggs, used to treat bone weakness.	Least Concern	Balti Brokapa
*Muntiacus muntjak*Zimmermann, 1780 (Cervidae) (Mun.mun)	Kakkar (D) Keri (G,Ba) (Barking deer)	Flesh Skin	Flesh is cooked and consumed as food. Skin is employed for costume making.	-------------------------------	Least Concern	Gujjar Bakarwal Dogra
*Moschus cupreus* Linnaeus, 1758 (Moschidae) (Mos.cup)	Quostori * Quostori heren* Roose kat (K) (Musk Deer)	Flesh Mask	Musk is used for magico-religious use. Flesh is cooked and consumed as food.	Musk is mixed with saffron to form small pellets taken orally to increase virility.	Vulnerable	Gujjar Pahari Bakarwal Kashmiri
*Naemorhedus goral* Hardwicke, 1825 (Bovidae) (Nae.gor)	Gorl* (Grey Himalayan Goral)	Flesh	Flesh is cooked and consumed as food.	-------------------------------	Near Threatened	Gujjar Pahari Bakarwal Kashmiri
*Ochotona ladacensis*Günther, 1875 (Ochotonidae) (Och.lad)	Zabra (Br) Zaabra (C) (Ladak pika)	Flesh	Flesh is cooked and consumed as food.	-------------------------------	Least Concern	Brokapa Changapa
*Ovis ammon* Linnaeus, 1758 (Bovidae) (Ovi.am)	Nyan (B) Neeyn (Br) Neeyaan (C) (Argali)	Flesh Horns	Flesh is cooked and consumed as food. Horns are used as trophies.	Flesh is cooked and consumed to overcome protein deficiency in adults.	Endangered	Balti Brokapa Changapa
*Ovis aries vignei* Blyth, 1841 (Bovidae) (Ovi.ari.vig)	Shapo (Br) Shap (B) Shaapo (C) (Urial)	Flesh Horns	Flesh is consumed as food. Horns are used as trophies.	Flesh is roasted and consumed to treat cough.	Vulnerable	Balti Brokapa Changapa
*Panthera pardus* Linnaeus, 1758 (Felidae) (Pan.par)	Chittra (G,Ba,P) Khas sae (K) Taandava (D) (Common Leopard)	Flesh Skin Claws Bones Whiskers	Skin is used in costume making and trophies, claws are boiled in water and used for bathing to overcome demonic possession. Whiskers burned to ashes are used as poison. Flesh made into amulets and used to ward off evil.	Traditionally bones are used for making medicine to treat cancer. Whiskers are made into amulets to treat asthma.	Vulnerable	Gujjar Pahari Bakarwal Kashmiri Dogra
*Pantholops hodgsonii* Abel, 1826 (Bovidae) (Pan.hod)	Rtsos (C) Resato (Br) (Tibetan antelope)	Flesh Horns Fur	Small part of the horn is tied around the arm to ease childbirth. Horns are also used as trophies. Flesh is cooked and consumed as food. Fur (Shahtoosh) is used to make luxury shawls.	-------------------------------	Near Threatened	Brokapa Changapa
*Panthera uncia* Schreber, 1775 (Felidae) (Pan.unc)	Kha-shae (K) Barfaani-chittra (G,P,Ba) Schan (Br) Schaan (B) (Snow Leopard)	Flesh Bones Skin Fur Bile	Sun dried flesh is made into amulets to ward against evil eye and demonic possessions. Bone and claw are employed as trophies. Skin and fur are used in costume making.	Bile is dried, mixed with lukewarm water, taken orally to treat respiratory disorders.	Vulnerable	Bakarwal Kashmiri Balti Brokapa Changapa
*Procapra picticaudata* Hodgson, 1846 (Bovidae) (Pro.pic)	Goa (Br) Goaa (B) Ga (C) (Tibetan gazelle)	Flesh Horns	Flesh is cooked and consumed as food.	Hornsare burned to ash and given orally in small quantities to treat diarrhea.	Near Threatened	Balti Brokapa Changapa
*Pseudois nayaur* Hodgson, 1833 (Bovidae) (Pse.nay)	Napo (Br) Naayo (B) Nemuv(C) (Bharal/Blue Sheep)	Flesh Horn Hair	Hair is used as a poisoning agent. Flesh is cooked and consumed as food.	For fast healing of wounds, horns are powdered and kept over the wound, then covered with cotton cloth.	Least Concern	Balti Brokapa Changapa
*Ursus thibetanus* Blandford 1888 (Ursidae) (Urs.thi)	Khrun haput (K) Reich (G,Ba,P) (Himalayan black bear)	Fat Bile Skin	-------------------------------	Shade-dried bile is taken orally with lukewarm water to treat jaundice, hemorrhoids, epilepsy, inflammation, and liver disorders. Skin is used as bedding for the paralyzed person. Fat (*haapat charab*) is rubbed on the body especially joints to treat joint pain and keep the body warm.	Vulnerable	Gujjar Pahari Bakarwal Kashmiri
*Semnopithecus schistaceus* Hodgson, 1840 (Cercopithecidae) (Sem.sch)	Langur (G,Ba,P,K) (Himalayan langur)	Flesh Tooth Nails	Nails are kept below the pillow to overcome bad dreams.	Flesh cooked is used to treat erectile dysfunction. Tooth is powdered very finely, and poured into the eye to treat cataract.	----------	Gujjar Pahari Bakarwal Kashmiri
*Macaca mulatta* Zimmermann, 1780 (Hylobatidae) (Mac.mul)	Puunz (K) (Monkey)	Bones	Some vertebras are used for black magic. In Hindu faith, *Macaca mulatta* is treated as sacred, believed to be the incarnation of deity *Hanuman* (avatar of Lord Shiva).	Bones are boiled; a glittering layer above the boiling water is collected and given orally to treat paralysis.	Least Concern	Kashmiri Dogra Gujjar
*Ursus arctos* Linnaeus, 1758 (Ursidae) (Urs.arc)	Wazul haaput (K) Denmo (Br) Dem (B) Dee (C) Rata reich (G,Ba,P) (Brown bear)	Fat Bile Skin Fur	Skin and fur is used in costume making.	Fat is rubbed on the joint to treat pain, bile is shade-dried and taken with lukewarm water in small quantities to treat gout, asthma, paralysis, tuberculosis, cough, pneumonia, and pulmonary affliction. Skin and fur are used as bedding for the paralyzed person.	Least Concern	Gujjar Pahari Bakarwal Kashmiri Balti Brokapa Changapa
*Vulpes ferrilata* Hodgson, 1842 (Canidae) (Vul.fer)	Watsay (B) Watssi (Br) (Tibetan Sand Fox)	Flesh Bones	Fur is used in costume making. Tail is used as a trophy.	Bones boiled in water to produce viscous fluid, taken orally to treat lung ulcers. Roasted flesh is used to cover back pain and rheumatic pain.	Least Concern	Balti Brokapa
*Vulpes vulpes* Linnaeus, 1758 (Canidae) (Vul.vul)	Laash (K) Lumdai (G,Ba,P) Watsay (Br) Watsay (B) (Fox)	Flesh Fur Tail	Dried flesh is tied to the arm to ward off evil. Tail is used as a trophy and fur for making hand gloves and other decorative purposes.	Flesh is cooked and consumed to treat leprosy.	Least Concern	Gujjar Pahari Kashmiri Balti Brokapa
*Rattus pyctoris* Hodgson,1845 (Muridae) (Rat.pyc)	Voyi (B) (Rat)	Faecal Pellets	In Hindu faith, *Rattus pyctoris* is treated as sacred, believed as the vehicle of the deity *Ganaish* (son of Lord Shiva)	Pellets are given orally to treat patients with urine issues.	Least Concern	Balti
Wild birds						
*Anser indicus* Latham, 1790 (Anatidae) (Ans.ind)	Nyagar (B) (Bar-headed Goose)	Flesh Eggs	Flesh and eggs are cooked and consumed as food.	-------------------------------	Least Concern	Balti
*Alectoris chukar* J. E. Gray, 1830 (Phasianidae) (Ale.chu)	Chukar (G,P,Ba) Srhakpa (B,Br, C) (Chukar)	Flesh Fat	Flesh is cooked and consumed as food. Bird is also kept in a small cage for amusement.	Fat is boiled to produce pale yellow oil among which 1–2 drops are dropped inside the ear to treat pain. Flesh is cooked, believed to treat gout and to maintain virility. Soup obtained from flesh is used for the treatment of paralysis.	Least Concern	Gujjar Pahari Bakarwal Balti Brokapa Changapa
*Anas acuta*Linnaeus, 1758 (Anatidae) (Ana.acu)	Shakar batak (K) (Northern Pintail)	Flesh	Flesh is cooked and consumed as food.	-------------------------------	Least Concern	Kashmiri
*Anas crecca* Linnaeus, 1758 (Anatidae) (Ana.cre)	Kal neej (K) (Common Teal)	Flesh	Flesh is cooked and consumed as food.	-------------------------------	Least Concern	Kashmiri
*Anas poecilorhyncha* Forster, 1781 (Anatidae) (Ana.poe)	Lider choons-Batak (K) (Indian spot-billed duck)	Flesh	Flesh is cooked and consumed as food.	-------------------------------	Least Concern	Kashmiri
*Arborophila torqueola* Valenciennes, 1826 (Phasianidae) (Arb.tor)	Shakar (K) (Necklaced-hill Partridge)	Flesh	Flesh is cooked and consumed as food.	-------------------------------	Least Concern	Kashmiri
*Ardeola grayii* Sykes, 1832 (Ardeidae) (Ard.gra)	Shataan (K) (Indian Pond Heron)	Claws	It is associated with the tales that it is an incarnation of the devil. Claws are rarely used in black magic.	-------------------------------	Least Concern	Kashmiri
*Aythya ferina* Linnaeus, 1758 (Anatidae) (Ayt.fer)	Vazul kal Batuk (K) (Common Pochard)	Flesh Droppings	Flesh is cooked and consumed as food.	Dried dropping soaked in water are painted on feet to overcome foot burning.	Vulnerable	Kashmiri
*Aythya fuligula* Linnaeus, 1758 (Anatidae) (Ayt.ful)	Aech Ladder (K) Tasoki (Br) Tasoi (B) (Tufted Duck)	Flesh Fat Feathers	Flesh is cooked and consumed as food. Long feathers from wings are burned to produce smoke, which is used to overcome nightmares.	Fat is mixed with young willow bark (*Salix alba*, *Salix pycnostachya*) and painted on the neck to treat pain.	Least Concern	Kashmiri Balti Brokapa
*Aythya nyroca* Guldenstadt, 1770 (Anatidae) (Ayt.nyr)	Aech Safed (K) Krofoso (B) (Ferrugin ous Pochard)	Flesh	Flesh is cooked and consumed as food.	Young ones (whole young one) are cooked to soup, used to gain strength after delivery.	Near Threatened	Kashmiri Balti
*Columba leuconota* Vigors, 1831 (Columbidae) (Col.leu)	Kootar (K) Mikran (B) (Snow Pigeon)	Flesh Blood	Flesh is cooked, consumed as food.	Flesh is cooked and given to the patients suffering from asthma and paralysis. Fresh blood is given to the patients suffering from mild stroke.	Least Concern	Kashmiri Balti
*Columba livia* Gmelin, 1789 (Columbidae) (Col.liv)	Kabutar (G, P) Qatar (K) (Rock Pigeon)	Flesh Blood	Flesh is cooked and consumed as food. Live onesare used to perform black magic by locking an amulet in mouth or to a leg.	Flesh is cooked and given to the patients suffering from asthma and paralysis. Fresh blood is used to treat mild strokes.	Least Concern	Gujjar Pahari Kashmiri
*Columba rupestris* Pallas, 181 (Columbidae) (Col.rup)	Kubatur (G, P) Qatar (K) Mikran (Br) Meekrn (B) (Hill Pigeon)	Flesh Droppings	Flesh is cooked and consumed as food.	Dried droppings are mixed with water to form a paste which is applied to the areas with inflammation.	Least Concern	Gujjar Pahari Kashmiri Balti Brokapa
*Cygnus columbianus* Ord, 1815 (Anatidae) (Cyg.col)	Shah Aanz (K) (Bewick Swan)	Flesh	Flesh is cooked and consumed as food.	-------------------------------	Least Concern	Kashmiri
*Glaucidium radiatum* Tickell, 1833 (Strigidae) (Gla.rad)	Olu (G,B) Raat Mungur (K) Oolu (D) (Owl)	Gallbladder Fat Blood	Blood is used by magicians for black magic.	Gall bladder is sun dried, powdered, and added with powdered rice and pond water to form a paste which is applied around the eyes to increase eyesight. Urinary incontinence is treated by mixing the fat with *Adiantum venustum* (Gewvtheer) plant and taken orally. Feathers are burned to ash which is used topically to treat skin diseases.	Least Concern	Gujjar Bakarwal Kashmiri Dogra
*Athene noctua*Scopoli, 1769 (Strigidae) (Ath.noc)	Tso-ro-s (Br) So so (B) (Little Owl)	Flesh Blood	Blood and flesh are used by local magicians to perform black magic.	-------------------------------	Least Concern	Balti Brokapa
*Hirundo rustica* Linnaeus, 1758 (Hirundinidae) (Hir.rus)	Kataij (K) (Barn Swallow)	-----------	Treated as sacred in Muslim faith, believed to have protected the holy Kaba from invaders.	-------------------------------	Least Concern	Kashmiri
*Lerwa lerwa* Hodgson, 1833 (Phasianidae) (Ler.ler)	Teetar (G, P) (Snow Partridge)	Flesh	Flesh is cooked and consumed as food.	Roasted flesh, painted with the paste of honey and cinnamon and used to treat common cold.	Least Concern	Gujjar Pahari
*Lophophorus impejanus* Latham, 1790 (Phasianidae) (Lop.imp)	Vankukur (K) Jangli kukud (G, P, B) (Himalayan Monal)	Flesh Crest feather	Flesh is cooked and consumed as food. Crest feathers are highly valued and used decoratively. Some people believe they gain social status by wearing costumes with feathers. Bird is also kept in a small cage for amusement.	-------------------------------	Least Concern	Gujjar Pahari Bakarwal Kashmiri
*Lophura leucomelanos* Latham, 1790 (Phasianidae) (Lop.leu)	Jangali Kukur (K) (Kalij Pheasant)	Flesh	Flesh is cooked and consumed as food.	-------------------------------	Least Concern	Kashmiri
*Mareca penelope* Linnaeus, 1758 (Anatidae) (Mar.pen)	Meegail (K) Gobrakpa (Br) Gobrekpe (B) (Eurasian Wigeon)	Flesh Eggs	Flesh is cooked and consumed as food.	Egg shells are powdered, mixed with dates and milk, and taken orally to treat infertility in males.	Least Concern	Kashmiri Balti Brokapa
*Marecas strepera* Linnaeus, 1758 (Anatidae) (Mar.str)	Aabee batak (K) Jung Nagma (Br) (Gadwall)	Flesh	Flesh is cooked, eaten as food, and believed to increase body strength.	-------------------------------	Least Concern	Kashmiri Brokapa
*Netta rufina* Pallas, 1773 (Anatidae) (Net.ruf)	Vajaj Choons Batak (K) (Red-crested pochard)	Flesh	Flesh is cooked and consumed as food.	-------------------------------	Least Concern	Kashmiri
*Passer domesticus* Linnaeus, 1758 (Passeridae) (Pas.dom)	Chaer (K) (House Sparrow)	Flesh Blood	Flesh is cooked and consumed as food.	It is believed that soup obtained from female flesh keeps heart issues away. Blood is used with curd and salt to treat wounds.	Least Concern	Kashmiri
*Pavo cristatus* Linnaeus, 1758 (Phasianidae) (Pav.cri)	Moor (D) (Indian Peafowl)	Flesh Faeces Feathers	Flesh is cooked and consumed as food. In Hinduism, peafowl is treated as sacred, known as the vehicle of the deity *Kartakia* (Son of Lord Shiva). Feathers are used as decoration and believed to bring fortune and wealth. Birds are also watched for amusement because of their attractive plumage.	Faeces are painted on the forehead and feet to overcome fever.	Least Concern	Dogra
*Perdix hodgsoniae* Hodgson, 1857 (Phasianidae) (Per.hod)	Hosov (Br) (Tibetan Partridge)	Flesh	Flesh is cooked as food.	Flesh is cooked and consumed to increase sexual potential.	Least Concern	Brokapa
*Porphyrio poliocephalus* Latham, 1801 (Rallidae) (Por.pol)	Shakar (K) (Grey-headed Swamphen)	Flesh	Flesh is cooked as food.	-------------------------------	------------	Kashmiri
*Pucrasia macrolopha* G.R. Gray, 1841 (Phasianidae) (Puc.mac)	Takay de mugri (G,P,Ba) Kakov (K) (Himalayan Koklass)	Flesh Gizzard	Flesh is cooked as food.	Sun dried gizzard (outer covering) is powdered and taken with lukewarm water to treat dry cough.	Least Concern	Gujjar Pahari Bakarwal Kashmiri
*Spatula clypeata* Linnaeus, 1758 (Anatidae) (Spa.cly)	Shakar (K) (Northern Shoveller)	Flesh	Flesh is cooked as food.	-------------------------------	Least Concern	Kashmiri
*Streptopelia decaocto* Frivaldszky, 1838 (Columbidae) (Str.dec)	Kookil (K) (Eurasian Collared Dove)	Flesh	Flesh is cooked as food.	Roasted flesh with spices such as cinnamon and black pepper, used to treat asthma.	Least Concern	Kashmiri
*Tetraogallus himalayensis* G. R. Gray, 1843 (Phasianidae) (Tet.him)	Jangli murag (G,P,Ba) Congmao (Br) Congim (B) Cojo (C) (Himalayan Snowcock)	Flesh	Flesh is cooked as food.	Flesh is cooked and eaten to treat asthma and cough in children.	Least Concern	Gujjar Pahari Bakarwal Balti Brokapa Changapa
*Tetraogallus tibetanus* Gould, 1854 (Phasianidae) (Tet.tib)	Teok(B) Steook (Br) (Tibetan Snowcock)	Flesh Droppings	Cooked flesh is consumed as food.	Cooked flesh is consumed to treat paralysis. Dried droppings are mixed with pond water to form paste, which is applied to the affected area to treat inflammation.	Least Concern	Balti Brokapa Changapa
*Tragopan melanocephalus* Gray, 1829 (Phasianidae) (Tra.mel)	Vankukud (G,Ba) Jangli murgi (P) (Western Tragopan)	Flesh	Cooked flesh is consumed as food.	Cooked flesh is a strong blood purifier.	Vulnerable	Gujjar Pahari Bakarwal
*Milvus migrans* Boddaert, 1783 (Accipitridae) (Mil.mig)	Gaant (K) (Kite)	Eyes Feathers Blood	Eyes, feathers, and blood are used in black magic.	-------------------------------	Least Concern	Kashmiri
Domestic mammals						
*Bos grunniens* Linnaeus, 1766 (Bovidae) (Bos.gru)	Dri (Br) Nak (B) Neak (C) (Yak)	Flesh Milk Trotters Skin Fur Dung	Milk and cooked flesh are consumed as food, skin and fur are used in costumes and bedding. Trotters are cooked and eaten to increase body strength. Dried dung is used as fuel.	Trotters are cooked and eaten to overcome arthritis.	----------	Balti Brokapa Changapa
*BostTaurus* Linnaeus, 1758 (Bovidae) (Bos.tau)	Gaav (K) Gaan (G, Ba, P) Haav (B) Haaso (Br) (Cow)	Flesh Milk Large intestines Fat	Milk, cooked flesh, and trotters are used as food. Dung is decomposed in a pit to get fertilizer. Cow is treated as sacred in the Hindu faith.	Large intestines are rubbed or covered on feet to treat chilblains. Fat from the abdominal cavity is massaged on the head and cracked heels before sleep to treat dandruff and smoothen the cracked heels. Intestines are used by traditional nurses (Daai) to help in parturition during delivery.	-----------	Gujjar Pahari Bakarwal Kashmiri Balti Brokapa
*Bos taurus primigenius* Bojanus, 1827 (Bovidae) (Bos.tau.pri)	Bail (G,Ba) Daand (K, P) Soov (B) Sovai (Br) (Ox)	Flesh Trotters Spleen Bones Dung	Flesh and trotters are cooked and eaten to increase body strength. Bones are decomposed to make fertilizers. Ox is also used in ploughing agricultural fields and is treated sacred in the Hindu faith.	Trotters are also eaten to maintain fluids in joints. Roasted spleen is used to treat coughs. Dung is painted on the untreated wound to remove larvae.	-----------	Gujjar Pahari Bakarwal Kashmiri Balti Brokapa
*Bubalus bubalis* Linnaeus, 1758 (Bovidae) (Bub.bub)	Baains (G,Ba) Meeins (D) Moonsh (K) Bhains (D) (Buffalo)	Flesh Trotters	Milk and cooked flesh are used as food. Trotters cooked without oil are consumed to increase body strength. Fat is used to reduce swelling and increase sexual potential.	-------------------------------	----------	Gujjar Pahari Bakarwal Dogra
*Camelus bactrianus* Linnaeus, 1758 (Camelidae) (Cam.bac)	Amoo (B) Emo (Br) (Double hump)	Flesh Milk	Meat is cooked as food and to gain body strength and vitality.	Meat is cooked and consumed to relieve joint pain. Milk is used as sexual stimulant and antidote.	----------	Balti Brokapa
*Camelus dromedarius* Linnaeus, 1758 (Camelidae) (Cam.dro)	Uhunt (P) Oont (K) (Camel)	Flesh Milk	Meat is cooked as food and to gain body strength and vitality.	Meat is cooked and consumed to relieve joint pain. Milk is used as sexual stimulant and antidote.	----------	Pahari Kashmiri
*Capra hircus* Linnaeus, 1758 (Bovidae) (Cap.hir)	Bakri (G,P,Ba) Kat (D) Chavaj (K) Roii (Br) Roei (B) Soaa (C) (Goat)	Milk Flesh Trotters Hair Hide Faecal pellets	Milk is consumed as food. Flesh is cooked and consumed as food, trotters are cooked and eaten to increase body strength. Body hair is used in costume sand bedding. Hide is used for praying by Muslims. Pellets are treated as best fertilizers for apple orchids, also believed to stop diseases in said orchids.	Milk is consumed to sharpen memory, lower body heat, and to treat stomach ulcers. Trotters are cooked and eaten to maintain fluids in the joints. Soup obtained from the brain is used to treat paralysis.	----------	Gujjar Pahari Bakarwal Kashmiri Dogra Balti Brokapa Changapa
*Capra aegagrus hircus* Linnaeus, 1758 (Bovidae) (Cap.aeg)	Changthangi (C) (Pashmina goat)	Milk Flesh Hair	Milk is consumed as foodand to sharpen memory. Flesh is cooked and consumed as food, body hair (pashmina) is used in costumes. Shawls are made from this pashmina, which have a very high price on the international market.		----------	Changapa
*Equus africanus asinus* Linnaeus, 1758 (Equidae) (Equ.afr)	Kahoot (G,Ba) Gada (G, Ba) (Donkey)	Urine	Used as a beast of burden.	Fresh urine collected early morning is used to treat dermatitis.	----------	Gujjar Bakarwal
*Equus ferus caballus* Linnaeus, 1758 (Equidae) (Equ.fer.cab)	Gur (K) Qooda (G,Ba, P) Losai (B) Sore (Br) Goday (C) (Horse)	Hair Urine Dung	Horses are used to carry goods and people. In rare cases, some local people use them in horse races.	Fresh urine collected early morning is used to treat dermatitis. Dung is painted over the wound to extract the larvae of pathogens. Hair from the tail is used to cut cysts developed on the skin.	----------	Gujjar Pahari Bakarwal Kashmiri Balti Brokapa Changapa
*Ovis aries* Linnaeus, 1758 (Bovidae) (Ovi.ari)	Paroo (G, Ba) Baid (P) Kath (K) Qursi (B) Sewq (C) Sew (Br) (Sheep)	Flesh Trotters Hair Faecal pellets Hide	Flesh is cooked and eaten as food. Soup obtained from trotters is used to gain strength. Wool is used in costumes. Liver, kidneys, head, and tongue are used in black magic. Hide is used for praying by Muslims. Fat is used in local recipes such as Wazwaan. Pellets are treated as best fertilizers for apple orchids, also believed to stop diseases in said orchids.	-------------------------------	----------	Gujjar Pahari Bakarwal Kashmiri Balti Brokapa Changapa
Domestic birds						
*Anser anser domesticus* Linnaeus, 1758 (Anatidae) (Ans.ans.dom)	Aanz (K) Enz (G) Naganpa (B) (Greylag Goose)	Flesh Eggs	Flesh and eggs are cooked and eaten as food.	Egg shells are powdered, taken with milk, used to increase sexual stamina.	----------	Gujjar Kashmiri Balti
*Gallus gallus domesticus* Linnaeus, 1758 (Phasianidae) (Gal.gal.dom)	Kukud (G, P) Kukudr(K) (Red Jungle Fowl)	Flesh Eggs	Flesh is cooked and eaten as food. Eggs are given to children for growth.	Flesh is cooked and eaten to increase libido and sexual power.	----------	Gujjar Pahari Kashmiri
*Anas platyrhynchos domesticus* Linnaeus, 1758 (Anatidae) (Ana.pla.dom)	Batak (K) Muru (B) (Domestic duck)	Flesh	Flesh is cooked and eaten as food.	Roasted flesh is used to increase virility and libido.	----------	Kashmiri Balti

* quoted with similar phytonym among all the studied groups, Br: Brokapa, B: Balti, C: Changapa, G: Gujjar, Ba: Bakarwal, P: Pahari, K: Kashmiri, D: Dogra. These letters are attached to the local names in the column depicting the local name spoken by respective ethnic group.

**Table 3:** Overlap of species usage across the selected ethnic groups.

Ethnic Groups	Number of Commonly Used Species	Name of Commonly Used Species
Bakarwal, Balti, Brokapa, Changapa, Dogra, Gujjar, Kashmiri, Pahari	1	*Capra hircus*
Bakarwal, Balti, Brokapa, Changapa, Gujjar, Kashmiri, Pahari	2	*Ovis aries*, *Ursus arctos*
Bakarwal, Balti, Brokapa, Dogra, Gujjar, Kashmiri. Pahari	1	*Panthera uncial*
Bakarwal, Balti, Brokapa, Gujjar, Kashmiri, Pahari	2	*Tetraogallus himalayensis*, *Equus ferus caballus*
Bakarwal, Brokapa, Changapa, Gujjar, Kashmiri, Pahari	1	*Capra sibirica hemalayanus*
Bakarwal, Balti, Brokapa, Changapa, Gujjar, Kashmiri	1	*Canis lupus*
Bakarwal, Dogra, Gujjar, Kashmiri, Pahari	2	*Panthera pardus*, *Ursus thibetanus*
Balti,Brokapa, Gujjar,Kashmiri, Pahari	3	*Columba rupestris*, *Bos taurus*, *Vulpes vulpes*
Bakarwal, Gujjar, Kashmiri, Pahari	6	*Moschus moschiferus*, *Capra falconeri*, *Semnopithecus schistaceus*, *Cervus elaphus hanglu*, *Lophophorus impejanus*, *Lophura leucomelanos*
Bakarwal, Dogra, Gujjar, Pahari	2	*Pavo cristatus*, *Boselaphus tragocamelus*
Balti, Brokapa, Gujjar, Pahari	1	*Alectoris chukar*
Bakarwal, Dogra, Gujjar, Kashmiri	1	*Glaucidium radiatum*
Bakarwal, Balti, Brokapa, Gujjar	1	*Lepus oiostolus*
Gujjar, Kashmiri, Pahari	4	*Hirundo rustica*, *Bos taurus primigenius*, *Gallus gallus domesticus*, *Columba livia*
Bakarwal, Gujjar, Pahari	3	*Pucrasia macrolopha*, *Naemorhedus goral*, *Tragopan melanocephalus*
Balti, Gujjar, Kashmiri	1	*Anser anser domesticus*
Bakarwal, Dogra, Gujjar	2	*Muntiacus muntjak*, *Hemitragus jemlahicus*
Balti, Brokapa, Kashmiri	1	*Aythya fuligula*
Bakarwal, Brokapa, Changapa	1	*Lynx lynx isabellinus*
Balti, Brokapa, Changapa	5	*Procapra picticaudata*, *Marmota caudata*, *Bos grunniens, Ovis ammon*, *Pseudois nayaur*
Brokapa, Changapa, Dogra	1	*Pantholops hodgsonii*
Gujjar, Pahari	2	*Lerwa lerwa*, *Bubalus bubalis*
Bakarwal, Gujjar	1	*Equus africanus asinus*
Kashmiri, Pahari	2	*Passer domesticus*, *Camelus dromedaries*
Balti, Kashmir	5	*Anas platyrhynchos domesticus*, *Anas acuta*, *Anas crecca*, *Columba leuconota*, *Aythya nyroca*
Dogra, Kashmiri	1	*Macaca mulatta*
Balti, Brokapa	7	*Athene noctua*, *Marmota himalayana*, *Tetraogallus tibetanus*, *Ovis aries vignei*, *Camelus bactrianus*, *Vulpes ferrilata*, *Lutra lutra*
Balti, Dogra	1	*Rattus pyctoris*
Brokapa, Changapa	1	*Ochotona ladacensis*
Kashmiri	13	*Porphyrio poliocephalus*, *Hystrix indica*, *Streptopelia decaocto*, *Ardeola grayii*, *Cygnus columbianus*, *Milvus migrans*, *Netta rufina*, *Anas poecilorhyncha*, *Spatula clypeata*, *Mareca penelope*, *Arborophila torqueola*, *Mareca strepera*, *Aythya farina*
Balti	2	*Anser indicus*, *Perdix hodgsoniae*
Changapa	2	*Capra aegagrus hircus*, *Cuon alpines*

## Data Availability

All the obtained data is provided in the research article.
